# Analysis of influencing factors and prediction model for fatigue among medical staff at a large primary centralized medical observation point

**DOI:** 10.3389/fpubh.2025.1722353

**Published:** 2026-01-12

**Authors:** Yingna Qu, Shuguang Zheng, Ting Yang, Lini Zheng, Fen Jiang, Fei Yang

**Affiliations:** Lanxi People’s Hospital, Jinhua, Zhejiang, China

**Keywords:** centralized medical observation points, fatigue prediction, ecological systems theory, multivariate regression analysis, prediction model, ROC curve analysis

## Abstract

**Objective:**

Long-term closed-loop management and the demanding nature of work at centralized medical observation points can increase fatigue among medical staff, impair physical and mental health, and degrade the quality of medical services. This study aims to evaluate the factors influencing fatigue among medical personnel at a primary centralized medical observation point, including personal conditions and working environment factors.

**Methods:**

A total of 145 medical staff members from Lanxi People’s Hospital, all of whom participated in epidemic prevention work at centralized medical observation points from January 2021 to April 2023, were enrolled in this study. Data were analyzed using a binary logistic regression model to evaluate predictive efficacy, with the study design grounded in the ecological systems theory framework.

**Results:**

The proportions of night shifts, participation in physical exercise, and gender were detected as independent factors associated with fatigue (all *p* < 0.05). The multivariate logistic regression model demonstrated higher predictive accuracy than the univariate model based on the night-shift proportion, indicating that the combined diagnostic model is valuable for diagnosing fatigue among medical staff.

**Conclusion:**

The proportion of night shifts, participation in physical exercise, and gender affect the occurrence of fatigue, and the combined diagnostic model predictive value is beneficial to the diagnosis of fatigue, thereby providing a basis for hospitals to develop targeted fatigue prevention strategies in night shift settings, personnel allocation, and increasing stress reduction measures.

## 1 Introduction

The centralized medical observation point is a control measure implemented for close contacts of confirmed cases, inbound personnel, and people from medium and high-risk areas during the COVID-19 prevention and control period. It offers several advantages, including sufficient space, high capacity for quarantined individuals, a semi-open working environment, and adequate natural ventilation in individual rooms. According to the Zhejiang Provincial Regulations on Epidemic Prevention and Control (1), all medical staff at these observation sites must adhere to closed-loop management protocols during their assignments. Specifically, high-risk individuals are directly transferred to quarantine facilities upon entry, and staff must wear protective clothing at all times when working in contaminated areas.

However, long-term closed-loop management, multiple processes of dressing isolation gowns (2), and the high intensity of work can lead to increased fatigue among medical staff, resulting in excessive energy consumption. Therefore, enhancing personnel management in science, optimizing the work environment at isolation points, and reducing fatigue are crucial to enabling county-level primary medical institutions to fulfill their essential roles in grassroots primary diagnosis efficiently. This optimization has become a key measure in the fight against epidemics.

Ecological systems theory (3) holds that the individual interacts with the environment, with the individual influencing the environment and the environment affecting the individual, a framework widely applied in the field of mental health (4–6). And the micro-systems are most important (7) in human development because they have direct effects on human development. In the special environment of a medical observation point, fatigue should be influenced by both personal and environmental factors.

However, most existing studies on fatigue among medical staff at centralized medical observation points have focused either on personal mental stress and psychological well-being (8, 9) or on the impact of the working environment (10–12). Notably, these studies rarely examine the interactions among modifiable real-world factors, such as working environment conditions and personnel management practices, that influence fatigue. Unfortunately, these researchers often overlook the interaction between realistic and controllable factors, such as the work environment and the management of medical personnel’s conditions.

This study has four aims: (1) to investigate factors influencing fatigue among medical staff under closed-loop management at a large-scale centralized medical observation point; (2) To evaluate the effects of personal conditions and working environment factors on fatigue; (3) To discover key fatigue-related factors using binary logistic regression, and (4) to develop a predictive model to inform strategies for fatigue prediction and mitigation. Personal conditions within the “microsystem” include gender, educational background, and physical activity. In contrast, the working environment factors in the “meso-system” include the proportion of night shifts, job categories, and the nature of isolation sites, regarding medical staff fatigue as a systemic outcome.

## 2 Methods

### 2.1 Study subjects

From January 2021 to April 2023, 145 medical staff members from Lanxi People’s Hospital who participated in epidemic prevention and control at centralized medical observation points were enrolled. Of these, 140 completed valid questionnaires, three were excluded due to missing data, and two due to incorrect completion, resulting in a valid response rate of 96.6%. Inclusion criteria were primary care medical staff; engagement in work at large-scale shelter-type centralized medical observation points; and completion of a closed-loop management cycle of at least 21 days (i.e., 14 days of on-site duty plus 7 days of post-duty quarantine, ≥14 + 7 days). Exclusion criteria were pre-existing physical illness, pre-existing fatigue before initiating closed-loop management, and use of psychotropic medications. The human ethics for this study was approved by the Lanxi People’s Hospital Human Ethics Committee, with the approval number 2022-KY-030.

### 2.2 Study questionnaire

Participants were recruited voluntarily at the observation point. After obtaining written informed consent from each participant, data were collected via an online questionnaire administered immediately after medical staff completed their shift handover, with collection overseen by daily duty personnel. The questionnaire was designed to collect basic information from all staff involved in epidemic prevention and control. It focused on the following areas: personal condition and working environment. Personal conditions include gender, age, educational background, marital status, whether the individual is an only child, participation in physical exercise (13, 14), and health status. Participation in physical exercise was defined as engaging in moderate-intensity exercise for ≥30 min per session, ≥3 times per week during the closed-loop period; alternatively, a total of ≥150 min of moderate-intensity exercise or ≥75 min of high-intensity exercise per week during the closed-loop period.

To assess health status, participants were evaluated using the Multi-Organ Symptom or Disease Score (MOSDS) questionnaire, administered after completing the closed-loop cycle (≥14 + 7 days). The expert medical group developed the MOSDS, which is intended to assess specific symptoms or disease states. The MOSDS consists of eight categories: respiratory, digestive, cardiovascular, eyes, ears, nose and throat, skin, neuropsychiatric, urinary, and oral cavity. Targeted diagnosis of specific symptoms and diseases reported in the early stage is conducted, and a workable rating scale is developed. One point is assigned to having symptoms corresponding to one of these categories, but not affecting work or sleep, and two points are assigned to having one type of symptom that has also affected work or sleep. The minimum score is zero points, and the maximum score is 16 points. Additionally, the MOSDS in this study was an unvalidated tool developed for rapid situational assessment in this specific crisis context, and its results should be considered exploratory.

Working environment factors included workplace category, type of isolation point, job rank, professional role, proportion of night shifts, and number of previous deployments to isolation points (15–18). Night shift proportion refers to the ratio of the total night shift hours to the total working hours during the epidemic prevention period.

### 2.3 Fatigue assessment

The Multidimensional Fatigue Inventory-20 (MFI-20) (19, 20) was used to assess four dimensions of fatigue: physical fatigue, mental fatigue, reduced motivation, and reduced activity. The Chinese version of the MFI-20 was used to evaluate fatigue comprehensively. A total score of ≥60 (21, 22) indicates significant fatigue symptoms, warranting further assessment and intervention.

Physical fatigue is defined as soreness and tiredness following excessive physical exertion; mental fatigue is exhaustion resulting from prolonged, intense cognitive work; psychological fatigue is low mood triggered by environmental changes; and pathological fatigue is tiredness and weakness caused by an underlying medical condition. Scores on the MFI-20 range from 0 to 100; higher scores indicate more severe fatigue. The assessment is an online questionnaire completed immediately when medical staff hand over their shifts, collected by the daily duty personnel assigned to carry out the work. Based on MFI-20 scores upon completion of the closed-loop cycle (≥14 + 7 days), the 145 participants were divided into two groups: the “non-fatigue group” (*n* = 93, MFI-20 < 60) and the“obvious fatigue group” (*n* = 47, MFI-20 ≥ 60). The validity of MFI-20 was verified using the KMO and Bartlett tests. The KMO value is 0.892, which is greater than 0.8. The research data are very suitable for extracting information, which reflects that the validity is good.

### 2.4 Statistical methods

Data analysis was conducted using SPSS 26.0. Categorical data were expressed as counts or percentages, and the Chi-squared test was utilized for group comparisons. As none of the measures followed a normal distribution (Shapiro–Wilk test, data not shown), the data are presented as median (interquartile range) “M (Q1, Q3).” For unordered categorical variables, the Pearson chi-squared test was used; for ordinal variables compared across multiple groups, the Kruskal–Wallis *H* test was applied. The nonparametric rank-sum test was employed for measured data. Multivariable logistic regression analysis was performed to identify factors associated with fatigue. Meaningful variables were used to establish a statistical diagnostic model, enabling the calculation of predicted values (Pre). Receiver operating characteristic (ROC) curve analysis was conducted to evaluate the model’s discrimination capability (23–25), and the Area Under the Curve (AUC) assessed the accuracy of the prediction model. A *p*-value of less than 0.05 (*p* < 0.05) was considered statistically significant.

## 3 Results

### 3.1 Data statistics

In this study, 145 medical staff members were surveyed, with 140 completing the questionnaire, yielding a response rate of 96.6%. Regarding personnel composition: 36 were doctors (35.7%), 74 were nurses (52.8%), 19 were medical technicians, and 11 were administrative personnel. Doctors and nurses together accounted for 88.5% of the total staff, highlighting their critical role in epidemic prevention at the observation point. In terms of education: 29 staff (20.7%) held specialist diplomas, 105 (75.0%) held bachelor’s degrees, the most common educational attainment, and the remaining 6 staff held higher degrees. For the nature of the isolation points, 81 medical staff worked in hotels, accounting for 57.8%, while 59 were stationed in shelters, factories, or schools, representing 42.2%.

Based on MFI-20 scales, 93 participants (66.4%) scored <60, classified as non-fatigue (66.4%). In contrast, 47 (33.6%) scored ≥60, indicating fatigue (33.6%). The results of the univariate analysis are displayed in Table 1.

**Table 1:** Univariate analysis of possible risk factors for fatigue.

Factor		Non-fatigue group (*n* = 93)	Fatigue group *n* = 47	$\chi^{2}/Z$	*P*
Personal condition					
Gender	Male	11 (11.8%)	17 (36.2%)	11.563	0.001
	Female	82 (88.2%)	30 (63.8%)		
Age		32 (27, 41)	31 (26, 38)	−0.546	0.585
Marital status	Unmarried	52 (55.9%)	30 (63.8%)	0.806	0.369
	Married	41 (44.1%)	17 (36.2%)		
Educational background	Secondary vocational school education	2 (2.2%)	2 (4.3%)	0.005	0.944
	Specialty graduates	20 (21.5%)	9 (19.1%)		
	Undergraduates	70 (75.3%)	35 (74.5%)		
	Master’s degree graduates	1 (1.1%)	1 (2.1%)		
Only children in the family	True	22 (23.7%)	14 (29.8%)	0.614	0.433
	False	71 (76.3%)	33 (70.2%)		
Participation in physical exercise	True	27 (29.0%)	22 (46.8%)	4.337	0.037
	False	66 (71.0%)	25 (53.2%)		
MOSDS questionnaire score		0 (0, 2)	0 (0, 2)	−0.075	0.940
Working environment					
Working place category	General Hospital	72 (77.4%)	33 (70.2%)	0.865	0.352
	Hospital Branch	21 (22.6%)	14 (29.8%)		
Nature of the isolation point	Cabins	22 (23.7%)	11 (23.4%)	0.128	0.938
	Hotel or guesthouse	53 (57.0%)	28 (59.6%)		
	Factories or schools	18 (19.4%)	8 (17%)		
Work category	Unrated	7 (7.5%)	7 (14.9%)	0.528	0.467
	Junior	42 (45.2%)	19 (40.4%)		
	Intermediate	32 (34.4%)	16 (34.0%)		
	Senior	12 (12.9%)	5 (10.6%)		
Professional category	Physician	23 (24.7%)	13 (27.7%)	1.431	0.709
	Nurse	52 (55.9%)	22 (46.8%)		
	Medical technology	11 (11.8%)	8 (17.0%)		
	Hospital administrative position	7 (7.5%)	4 (8.5%)		
The number of times the isolation point is.		1 (1, 2)	1 (1, 2)	−0.082	0.934
Night shift proportion (%)		27.8 (17.2, 39.4)	40 (18.5, 23.3)	−2.035	0.042

Gender distribution differed significantly between groups (χ^2^ = 11.563, *p* = 0.001): the non-fatigue group included 11 males (11.8%) and 82 females (88.2%), while the fatigue group included 17 males (36.2%) and 30 females (63.8%). The median proportion of night shifts relative to total working hours was significantly lower in the non-fatigue group (27.8%, IQR: 17.2–39.4%) than in the fatigue group (40.0%, IQR: 18.5–23.3%; *Z* = −2.035, *p* = 0.042).

In the non-fatigue group, 27 staff members (29.0%) participated in physical exercise, while 66 staff members (71.0%) did not. In the fatigue group, 22 staff (46.8%) participated in physical exercise, and 25 (53.2%) did not, indicating a significant difference at the 0.05 level (*p* = 0.037).

### 3.2 Multiple factors logistic regression analysis

Fatigue was the dependent variable (coded as 1 = obvious fatigue, 0 = no fatigue). Independent variables included three factors with *p* < 0.05 in univariate analysis: gender, proportion of night shifts, and participation in physical exercise. Gender and participation in physical exercise were treated as categorical variables (gender: 1 = male, 2 = female; participation in physical exercise: 0 = no, 1 = yes), and the proportion of night shifts was treated as a continuous variable. The Hosmer-Lemeshow (HL) test was used to assess model fit, with the null hypothesis that the model’s predicted values match observed values. The HL test result was non-significant (χ^2^ = 5.349, *p* = 0.720), indicating that the model fit the data well. The Nagelkerke *R*^2^ was 0.216, indicating that the model explained 21.6% of the variance in fatigue. The model’s overall predictive accuracy was 73.57%, which is considered acceptable. The VIFs for three indicators are 1.057, 1.061, and 1.028, close to 1, indicating that the existing predictors can be retained for subsequent analysis and that the model fits well.

The model formula is: In (p/1−p)= −0.761 + 0.029 × night shift proportion + 0.993 × participation in physical exercise −1.646 × gender (where p represents the probability of fatigue is 1, and 1−p represents the probability of fatigue is 0). The regression results (Table 2) indicate that the proportion of night shifts, participation in physical exercise, and gender are independent factors affecting fatigue, all with *p* values less than 0.05 (P<0.05) .

**Table 2:** Multivariate logistic regression analysis affecting fatigue.

Variable	*β*	Standard error	Wald	*P*	OR value	95% CI	VIF	HL test
Participation in physical exercise (0=false,1=true)	0.993	0.420	5.593	0.018	2.699	1.185–6.146	1.057	χ2=5.349 p=0.720
Night shift proportion	0.029	0.010	8.843	0.003	1.030	1.010–1.050	1.061	
Gender (1=male2=female)	−1.646	0.476	11.962	0.001	0.193	0.076–0.490	1.028	

Nagelkerke *R*^2^ = 0.216.

Proportion of night shifts: The regression coefficient was 0.029 (*p* = 0.003), with an OR of 1.030(95% CI: 1.010–1.050). This indicates that for each 1-percentage-point increase in the proportion of night shifts, the odds of fatigue increase by 3%.

Participation in physical exercise: The regression coefficient was 0.993 (*p* = 0.018), with an OR of 2.699 (95% CI: 1.185–6.146). This indicates that medical staff who engaged in regular physical exercise had 2.699 times higher odds of experiencing fatigue than those who did not (*p* < 0.05).

Gender difference: the regression coefficient was −1.646 (*p* = 0.001), with an OR of 0.193 (95% CI: 0.076–0.490). This indicates that female medical staff had 0.193 times the odds of experiencing fatigue compared to male staff (*p* < 0.01).

### 3.3 Model predicts diagnostic value

**Figure 1:**
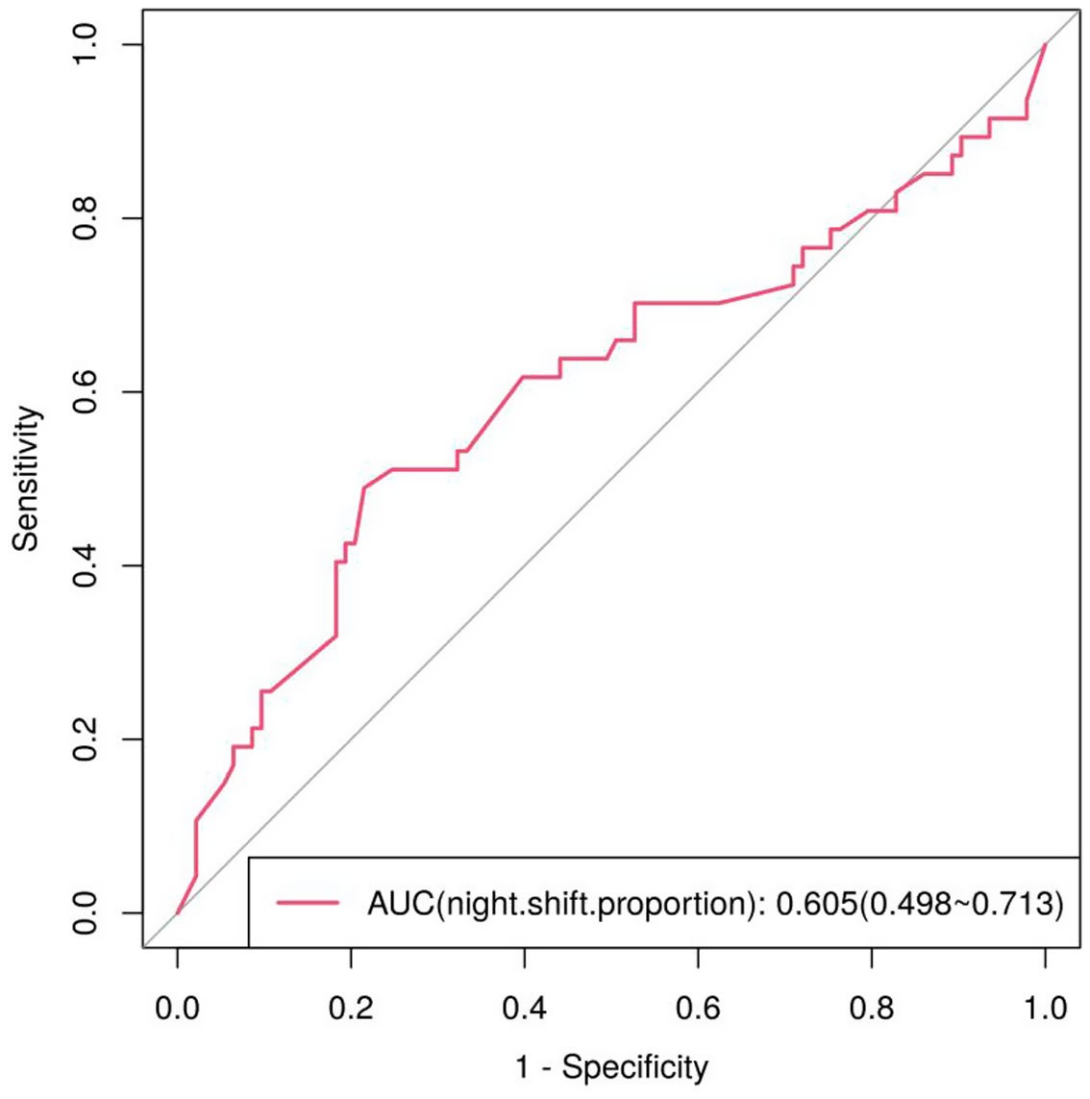
ROC curve for predicting fatigue as a percentage of the night shift.

The results of the univariate analysis suggest that the proportion of night shift hours in total working hours may be a risk factor for fatigue. A univariate ROC curve analysis was performed to evaluate the predictive value of night shift proportion alone. The analysis was significant (*p* = 0.042), with an AUC of 0.605 (95% CI: 0.499–0.712), indicating modest diagnostic utility for fatigue. Based on the ROC curve, the optimal cut-off value for the night shift proportion, according to the maximum Youden Index, was 40.83%, with a sensitivity of 48.9% and a specificity of 78.5% (Figure 1).

**Figure 2:**
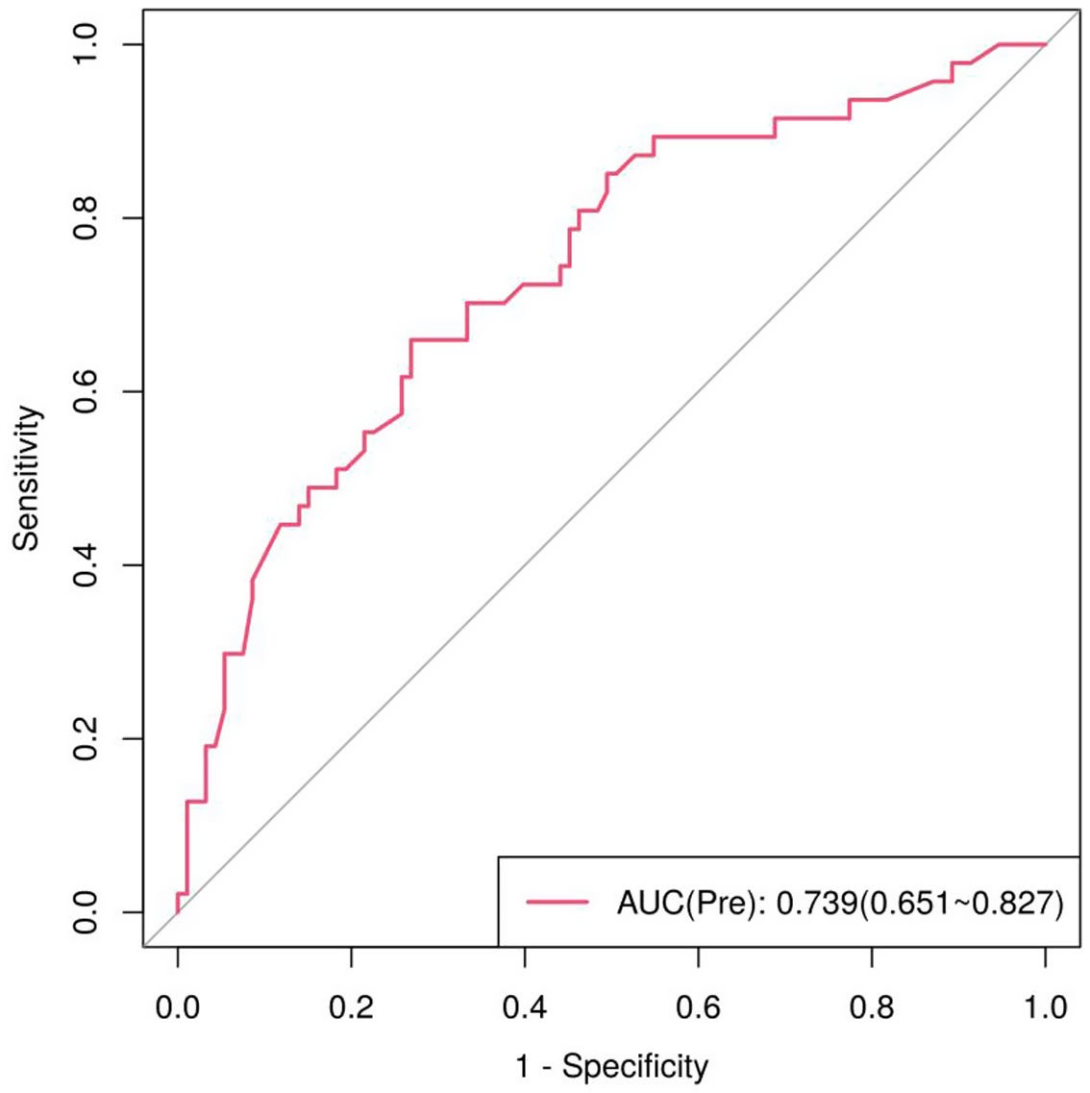
ROC curves of logistic regression model (Pre) for fatigue prediction.

For the multivariate model, we calculated the predicted probability of fatigue (Pre) using the three independent variables (participation in physical exercise, proportion of night shifts, and gender) and performed ROC curve analysis to evaluate its predictive performance. The ROC curve for the multivariate model was plotted using the logit-transformed predicted probability [logit(P)]. The analysis was highly significant (*p* < 0.001), with an AUC of 0.739 (95% CI: 0.651–0.827). AUC > 0.7 indicates that the multivariate logistic regression model has good predictive performance. The optimal cut-off value for the Youden Index was 0.325, with a sensitivity of 66.0% and a specificity of 73.1% (Figure 2).

## 4 Discussion

### 4.1 Main findings of this study

This study detected three independent factors associated with fatigue among medical staff at a large primary centralized medical observation point: proportion of night shifts, participation in physical exercise, and gender. Notably, the multivariate predictive model incorporating these three factors demonstrated higher diagnostic value for fatigue than the univariate model based on night-shift proportion alone.

### 4.2 What is already known on this topic

Existing studies on fatigue among medical staff at centralized medical observation points have primarily focused on two areas: the severity of fatigue among doctors and nurses (26, 27), with a focus on psychological factors (28–30); and the impact of working conditions (31, 32). Notably, few studies have integrated both personal and environmental factors into their analysis.

Previous studies have identified several predictors of fatigue among medical staff, including demographic variables (e.g., gender), clinical characteristics, and hospital-specific factors (e.g., night shift frequency), and physical exercise. Night shifts were recognized as predictors of fatigue among emergency department clinicians (33), and the main ICU factors influencing different potential categories of nurse alarm fatigue (34). Among studies assessing possible predictors of compassion satisfaction and compassion fatigue in health care workers, the most frequently studied predictors were age, gender, profession, and workload (35). In contrast, female gender emerged as a significant predictor of fatigue scores (36). Previous research has shown that nurses who engage in regular physical activity report lower burnout than those who are less active, suggesting that moderate exercise may mitigate fatigue (37). Similarly, Huibers et al. found a mild negative correlation between weekly exercise frequency and chronic fatigue (38).

### 4.3 What this study adds

This study builds on prior work by using binary logistic regression to analyze the combined effects of personal and work-environment factors on fatigue among medical staff at centralized medical observation points. Our findings confirm three independent factors associated with fatigue: proportion of night shifts, participation in physical exercise, and gender (all *p* < 0.05).

Furthermore, ROC curve analysis identified a diagnostic threshold of 40.83% for the night shift proportion, indicating that a higher proportion was associated with a higher likelihood of fatigue. This aligns with prior research linking night shifts to fatigue in medical staff (39). This finding has practical implications: when scheduling shifts at centralized medical observation points, hospitals should aim to limit night shifts.

In contrast to prior studies, our regression analysis found that medical staff who engaged in regular physical exercise had 2.699 times higher odds of fatigue than those who did not (OR = 2.699, 95% CI: 1.185–6.146) (40). A potential explanation for this discrepancy is that medical staff at centralized observation points work in high-intensity environments, where they are already prone to fatigue, burnout, and psychological anxiety (41). Adding regular exercise may further reduce rest time and increase physical strain, leading to exercise-induced fatigue, rather than the fatigue-mitigating effects observed in less demanding work settings. Nevertheless, an alternative explanation is that medical staff have already exhibited pre-symptomatic fatigue, or elevated stress may have adopted exercise as an intentional coping strategy. Consequently, this finding requires subsequent investigations utilizing longitudinal study designs and more detailed exercise metrics (including frequency, intensity, type, and duration).

The gender finding in our study that female staff had 0.193 times the odds of fatigue compared to male staff (OR = 0.193, 95% CI: 0.076–0.490) suggests that men are more susceptible to fatigue in response to stressful situations such as health events like the COVID-19 pandemic. Working at quarantine sites requires maintaining a high level of mental stress for extended periods and responding effectively to emergencies. There are indeed gender differences in stress management, especially for men (42, 43), who find it more challenging to cope with stressful events, which leads to fatigue.

ROC curve comparisons (Table 3) confirm that the univariate model using night shift proportion alone has limited predictive utility (AUC = 0.605, *p* = 0.042), while the multivariate model (Pre) demonstrates significantly better performance (AUC = 0.739, *p* < 0.001). This higher AUC, along with improved sensitivity (66.0% vs. 48.9%) and maintained specificity (73.1% vs. 78.5%), indicates substantial combined diagnostic value (44, 45) and confirms that the multivariate model enhances fatigue prediction accuracy.

**Table 3:** Comparison of ROC analysis results.

Predictive variable	AUC	Standard error	*p*	95% CI
Regression model of Pre	0.739	0.045	0.000**	0.651 ~ 0.827
Regression model of night shift proportion	0.605	0.054	0.042*	0.499 ~ 0.712

*p < 0.05, **p < 0.01.

### 4.4 Limitations of this study

This study has four main limitations. First, due to time constraints and spatial limitations during the COVID-19 pandemic, we were unable to include quantitative data (e.g., physical exercise, disease measurement, workload intensity) that may influence fatigue. Second, the sample size is limited to all participants from Lanxi People’s Hospital, limiting the generalizability of our findings to medical staff at other centralized observation points or in different regions. Future studies should involve broader participation and incorporate multiple centers across various regions. Third, we measured fatigue solely using the MFI-20 scale and MOSDS, which is unvalidated for medical staff fatigue and captures multidimensional, but not context-specific, fatigue (e.g., work-related vs. general fatigue), potentially limiting the depth of our fatigue assessment. Forth, while the model shows acceptable discrimination (AUC = 0.739) in this study, its stability and performance in new populations are unknown. Therefore, the model requires external validation in independent, multi-center cohorts before it can be considered for clinical or managerial application.

## 5 Conclusion

In conclusion, the multivariate logistic regression model developed in this study effectively enhances the diagnostic efficiency of fatigue among medical staff at centralized medical observation points. Combined evaluation of the three key variables: proportion of night shifts, participation in physical exercise, and gender, yields the highest diagnostic accuracy, providing hospitals with a valuable tool to implement targeted fatigue prevention measures.

Specifically, the work arrangements in centralized medical observation sites should consider the proportion of night shifts (46) within total working hours, opportunities for physical exercise, and gender factors. First, our model suggests that a higher proportion of night shifts is a significant risk factor for fatigue, and hospital management should reasonably limit night-shift duration to ensure sufficient rest (47, 48). Second, according to the work tasks, the proportion of male and female staff should be reasonably matched, taking into account both work efficiency and humanistic care, and psychological counseling can be provided. Third, the role of physical exercise requires further investigation with longitudinal designs and more detailed metrics. In addition, based on current research results, exercise is indeed associated with fatigue during the epidemic response, and some measures to mitigate exercise fatigue can be implemented. For example, medical staff should provide more physical relaxation, and the lounge can be equipped with massage devices and arrange online or on-site psychological counseling courses each week to relieve overly tense nerves and the body.

All of the measures would help medical staff at isolation points maintain their physical and mental energy amid the demands of their roles and respond efficiently to the responsibilities of centralized medical observation at the grassroots level (49, 50).

Our model suggests that a higher proportion of night shifts is a significant risk factor for fatigue, while the role of physical exercise requires further investigation with more detailed metrics.

## Data Availability

The original contributions presented in the study are included in the article/supplementary material, further inquiries can be directed to the corresponding author.

## References

[1] Health Commission of Zhejiang Province. Notice of the Health Commission of Zhejiang Province on Further Doing a Good Job in the Routine Prevention and Control of the Novel Coronavirus Pneumonia Epidemic. 2020. Available online at: https://wsjkw.zj.gov.cn/art/2020/5/21/art_1229560650_2320265.html (Accessed May 21, 2020).

[2] Jiang CT Chen DY Zhang R Ren F Zheng HW Yue Y . Improved comfort of medical protective clothing for medical staff facing high-intensity infectious diseases. Front Public Health. (2025) 13:1643043. doi: 10.3389/fpubh.2025.1643043 40860550 PMC12375584

[3] Navarro JL Tudge JRH. Technologizing Bronfenbrenner: neo-ecological theory. Curr Psychol. (2022):1–17. doi: 10.1007/s12144-022-02738-3 PMC8782219 35095241

[4] Vaezghasemi M Vogt T Lindkvist M Pulkki-Brännström AM Richter Sundberg L Lundahl L . Multifaceted determinants of social-emotional problems in preschool children in Sweden: an ecological systems theory approach. SSM Popul Health. (2023) 21:101345. doi: 10.1016/j.ssmph.2023.101345, 36785550 PMC9918800

[5] Ismail SM Khasawneh MAS Kakkad A Malathi H Dash A Chauhan AS. Cognitive-environmental coherence theory (CECT): a novel framework for culturally-embedded mental health. Asian J Psychiatr. (2025) 109:104536. doi: 10.1016/j.ajp.2025.104536, 40413931

[6] Liu X Ngoubene-Atioky AJ Yang X Deng Y Tang J Wu L . The effect of childhood family adversity on adulthood depression among Chinese older migrant workers: gender differences in the mediating role of social-ecological systems. BMC Public Health. (2024) 24:2005. doi: 10.1186/s12889-024-19397-7, 39061001 PMC11282819

[7] Feng L Zhang L Zhong H. Perceived parenting styles and mental health: the multiple mediation effect of perfectionism and altruistic behavior. Psychol Res Behav Manag. (2021) 14:1157–70. doi: 10.2147/prbm.S318446, 34377038 PMC8349536

[8] Arnold-Forster A Moses JD Schotland SV. Obstacles to physicians' emotional health - lessons from history. N Engl J Med. (2022) 386:4–7. doi: 10.1056/NEJMp2112095, 34979069

[9] Hadi B Mohammed SH. Impact of the COVID-19 pandemic on nurses mental health status in Iraq. J Educ Health Promot. (2022) 11:317. doi: 10.4103/jehp.jehp_637_22, 36438991 PMC9683455

[10] Aronsson G Theorell T Grape T Hammarström A Hogstedt C Marteinsdottir I . A systematic review including meta-analysis of work environment and burnout symptoms. BMC Public Health. (2017) 17:264. doi: 10.1186/s12889-017-4153-7, 28302088 PMC5356239

[11] Jin H Xiao M Gong Z Zhao Y. Influence of different protection states on the mental fatigue of nurses during the COVID-19 pandemic. Risk Manag Healthc Policy. (2022) 15:1917–29. doi: 10.2147/RMHP.S377936, 36268181 PMC9578785

[12] Cutshall SM Mallory MJ Noehl SM Soderlind JN Fischer KM Nanda S . Effect of aromatherapy on perceived mental health parameters for academic department workers working from home during the COVID-19 pandemic: a pilot study. Glob Adv Integr Med Health. (2024) 13:27536130241267748. doi: 10.1177/27536130241267748, 39070282 PMC11273579

[13] Latimer-Cheung AE Pilutti LA Hicks AL Martin Ginis KA Fenuta AM MacKibbon KA . Effects of exercise training on fitness, mobility, fatigue, and health-related quality of life among adults with multiple sclerosis: a systematic review to inform guideline development. Arch Phys Med Rehabil. (2013) 94:1800–28.e3. doi: 10.1016/j.apmr.2013.04.020 23669008

[14] Harrison AM Safari R Mercer T Picariello F van der Linden ML White C . Which exercise and behavioural interventions show most promise for treating fatigue in multiple sclerosis? A network meta-analysis. Mult Scler. (2021) 27:1657–78. doi: 10.1177/1352458521996002, 33876986 PMC8474304

[15] Richter K Acker J Adam S Niklewski G. Prevention of fatigue and insomnia in shift workers-a review of non-pharmacological measures. EPMA J. (2016) 7:16. doi: 10.1186/s13167-016-0064-4, 27486484 PMC4970219

[16] Min A Min H Hong HC. Work schedule characteristics and fatigue among rotating shift nurses in hospital setting: an integrative review. J Nurs Manag. (2019) 27:884–95. doi: 10.1111/jonm.12756, 30737987

[17] Kida R Takemura Y. Working conditions and fatigue in Japanese shift work nurses: a cross-sectional survey. Asian Nurs Res (Korean Soc Nurs Sci). (2022) 16:80–6. doi: 10.1016/j.anr.2022.03.001, 35304328

[18] Min A Kim YM Yoon YS Hong HC Kang M Scott LD. Effects of work environments and occupational fatigue on care left undone in rotating shift nurses. J Nurs Scholarsh. (2021) 53:126–36. doi: 10.1111/jnu.12604, 33205904

[19] Davis MP Walsh D. Mechanisms of fatigue. J Support Oncol. (2010) 8:164–74. 20822034

[20] Smets EM Garssen B Bonke B De Haes JC. The multidimensional fatigue inventory (MFI) psychometric qualities of an instrument to assess fatigue. J Psychosom Res. (1995) 39:315–25. doi: 10.1016/0022-3999(94)00125-o, 7636775

[21] Tian J Hong JS. Application of the Chinese version of the MFI-20 in detecting the severe fatigue in cancer patients. Support Care Cancer. (2013) 21:2217–23. doi: 10.1007/s00520-013-1783-x, 23503801

[22] Maass SWMC Brandenbarg D Boerman LM Verhaak PFM de Bock GH Berendsen AJ. Fatigue among long-term breast Cancer survivors: a controlled cross-sectional study. Cancer. (2021) 13:1301. doi: 10.3390/cancers13061301, 33803966 PMC8001130

[23] Helfenstein U Steiner M. The use of logistic discrimination and receiver operating characteristics (ROC) analysis in dentistry. Community Dent Health. (1994) 11:142–6. 7953932

[24] Chen L Xia S Lin Y Chen Y Xian L Yang Y . The role of coagulopathy and subdural hematoma thickness at admission in predicting the prognoses of patients with severe traumatic brain injury: a multicenter retrospective cohort study from China. Int J Surg. (2024) 110:5545–62. doi: 10.1097/JS9.0000000000001650 38752515 PMC11392125

[25] Lai H Gao K Li M Li T Zhou X Zhou X . Handling missing data and measurement error for early-onset myopia risk prediction models. BMC Med Res Methodol. (2024) 24:194. doi: 10.1186/s12874-024-02319-x, 39243025 PMC11378546

[26] Ruiz-Fernández MD Ramos-Pichardo JD Ibáñez-Masero O Cabrera-Troya J Carmona-Rega MI Ortega-Galán ÁM. Compassion fatigue, burnout, compassion satisfaction and perceived stress in healthcare professionals during the COVID-19 health crisis in Spain. J Clin Nurs. (2020) 29:4321–30. doi: 10.1111/jocn.15469, 32860287

[27] Pignatiello GA Tsivitse E O'Brien J Kraus N Hickman RL Jr. Decision fatigue among clinical nurses during the COVID-19 pandemic. J Clin Nurs. (2022) 31:869–77. doi: 10.1111/jocn.15939, 34291521 PMC8447365

[28] Sumner RC Kinsella EL. Grace under pressure: resilience, burnout, and wellbeing in frontline Workers in the United Kingdom and Republic of Ireland during the SARS-CoV-2 pandemic. Front Psychol. (2020) 11:576229. doi: 10.3389/fpsyg.2020.576229 33584412 PMC7874970

[29] Wang D Xie X Tian H Wu T Liu C Huang K . Mental fatigue and negative emotion among nurses during the COVID-19 pandemic. Curr Psychol. (2022) 41:8123–31. doi: 10.1007/s12144-022-03468-2, 35854701 PMC9285871

[30] Li Y Wang X Li M Hu B Cheng J Chen H . Factors associated with depression, anxiety, stress, PTSD, and fatigue of medical staff during the COVID-19 pandemic in Shanghai: a two-phase cross-sectional study. Braz J Med Biol Res. (2025) 58:e13943. doi: 10.1590/1414-431X2024e13943, 40053033 PMC11884776

[31] Zhang Y Xu Q Ma J Wang Z Lu S. Pandemic fatigue and clinical front-line medical staff health, job status during the COVID-19 pandemic: a cross-sectional survey after the lifting of epidemic restrictions. Nurs Open. (2024) 11:e2081. doi: 10.1002/nop2.2081, 38268297 PMC10782229

[32] Frenkel MO Pollak KM Schilling O Voigt L Fritzsching B Wrzus C . Stressors faced by healthcare professionals and coping strategies during the early stage of the COVID-19 pandemic in Germany. PLoS One. (2022) 17:e0261502. doi: 10.1371/journal.pone.0261502, 35041679 PMC8765664

[33] Alqahtani JS Arowosegbe A Oyelade T Aldhahir AM Alghamdi SM Alqarni AA . The effect of cumulative night shift duties on insomnia, fatigue, and mental health in intensive care unit. Heliyon. (2024) 10:e31066. doi: 10.1016/j.heliyon.2024.e31066, 38784539 PMC11112310

[34] Eldevik MF Flo E Moen BE Pallesen S Bjorvatn B. Insomnia, excessive sleepiness, excessive fatigue, anxiety, depression and shift work disorder in nurses having less than 11 hours in-between shifts. PLoS One. (2013) 8:e70882. doi: 10.1371/journal.pone.0070882, 23976964 PMC3744484

[35] Li Y Wang Y Lv X Li R Guan X Li L . Effects of factors related to shift work on depression and anxiety in nurses. Front Public Health. (2022) 10:926988. doi: 10.3389/fpubh.2022.926988, 35910870 PMC9326492

[36] Turchi V Verzuri A Nante N Napolitani M Bugnoli G Severi FM . Night work and quality of life. A study on the health of nurses. Ann Ist Super Sanita. (2019) 55:161–9. doi: 10.4415/ANN_19_02_08, 31264639

[37] Englund S Piehl F Kierkegaard M. High-intensity resistance training in people with multiple sclerosis experiencing fatigue: a randomised controlled trial. Mult Scler Relat Disord. (2022) 68:104106. doi: 10.1016/j.msard.2022.104106, 36037752

[38] Huibers MJ Leone SS van Amelsvoort LG Kant I Knottnerus JA. Associations of fatigue and depression among fatigued employees over time: a 4-year follow-up study. J Psychosom Res. (2007) 63:137–42. doi: 10.1016/j.jpsychores.2007.02.014, 17662749

[39] Li L Zhang W Chen Y. Exploring the factors influencing alarm fatigue in intensive care units nurses: a cross-sectional study based on latent profile analysis. PLoS One. (2025) 20:e0327644. doi: 10.1371/journal.pone.0327644, 40622939 PMC12233232

[40] Keightley P Reay RE Pavli P Looi JC. Inflammatory bowel disease-related fatigue is correlated with depression and gender. Austrl Psychiatry. (2018) 26:508–13. doi: 10.1177/1039856218772245, 29737197

[41] Guerrero K Fleming S Calderon A Fontenot N. Original research: addressing nurse burnout: the relationship between burnout and physical activity. Am J Nurs. (2024) 124:20–6. doi: 10.1097/01.NAJ.0001023020.53993.34, 38728132

[42] Baratta MV Gruene TM Dolzani SD Chun LE Maier SF Shansky RM. Controllable stress elicits circuit-specific patterns of prefrontal plasticity in males, but not females. Brain Struct Funct. (2019) 224:1831–43. doi: 10.1007/s00429-019-01875-z, 31028464 PMC6565440

[43] Assari S Lankarani MM. Stressful life events and risk of depression 25 years later: race and gender differences. Front Public Health. (2016) 4:49. doi: 10.3389/fpubh.2016.00049, 27047914 PMC4805579

[44] Yu F Somerville D King A. Exploring the impact of 12-hour shifts on nurse fatigue in intensive care units. Appl Nurs Res. (2019) 50:151191. doi: 10.1016/j.apnr.2019.151191, 31515156

[45] Takahashi K Uchiyama H Yanagisawa S Kamae I. The logistic regression and ROC analysis of group-based screening for predicting diabetes incidence in four years. Kobe J Med Sci. (2006) 52:171–80. 17329955

[46] Cook NF Boore JR. Managing patients suffering from acute and chronic fatigue. Br J Nurs. (1997) 6:811–5. doi: 10.12968/bjon.1997.6.14.811, 9283306

[47] Brown M Tucker P Rapport F Hutchings H Dahlgren A Davies G . The impact of shift patterns on junior doctors' perceptions of fatigue, training, work/life balance and the role of social support. Qual Saf Health Care. (2010) 19:e36. doi: 10.1136/qshc.2008.030734, 21127102 PMC3002836

[48] Josten EJ Ng ATJE Thierry H. The effects of extended workdays on fatigue, health, performance and satisfaction in nursing. J Adv Nurs. (2003) 44:643–52. doi: 10.1046/j.0309-2402.2003.02854.x, 14651687

[49] Corfield EC Martin NG Nyholt DR. Co-occurrence and symptomatology of fatigue and depression. Compr Psychiatry. (2016) 71:1–10. doi: 10.1016/j.comppsych.2016.08.004, 27567301

[50] Eriksen W Bruusgaard D. Do physical leisure time activities prevent fatigue? A 15 month prospectivestudy of nurses' aides. Br J Sports Med. (2004) 38:331–6. doi: 10.1136/bjsm.2002.004390, 15155438 PMC1724835

